# The role of Academic Health Centers in Transformative Medical Education

**DOI:** 10.6061/clinics/2019/e1466

**Published:** 2019-08-27

**Authors:** José Otávio Costa Auler Júnior, Talita de Almeida, Eduardo Moacyr Krieger

**Affiliations:** I Departamento de Cirurgia, Faculdade de Medicina FMUSP, Universidade de São Paulo, São Paulo, SP, BR; II Escritorio Internacional, Comissao de Relacoes Internacionais, Faculdade de Medicina FMUSP, Universidade de Sao Paulo, Sao Paulo, SP, BR; III Diretoria Executiva, Comissao de Relacoes Internacionais, Faculdade de Medicina FMUSP, Universidade de Sao Paulo, Sao Paulo, SP, BR

Academic Health Centers (AHC) were created in North America in the 20^th^ century, at a time when hospitals worldwide were utilized mostly as charitable housing for aged and chronically ill people. At that time, hospitals were not engaged in treatment and were not involved in medical education or research; moreover, there was no organized teaching or training of future doctors during the undergraduate period 1. The turning point in the transformation of these old health systems into modern medicine, including today's Academic Health Centers, occurred in 1910 when Abraham Flexner, a well-known educator, received a commission from the Institute for Advanced Study in Princeton to write a report about the state of American medical schools. The basis of Flexner's report, which required the American Medical Association to reform medical education, was the establishment of “requirements of higher standards of admission for applying medical students, a long period of undergrad training linked to clinical and basic research and high teaching quality in medical schools”. The report instigated a profound transformation of medical education in the USA in the following years and nurtured the development of Academic Health Centers over the next decades 2. Important medical discoveries, well-qualified doctors, focused research, and translational medicine, all linked to AHCs, flourished in many places, strengthening universities and affiliated hospitals and characterizing the “Golden Age” of medicine that followed the restructuring of medical education in the USA 2.

Although Academic Health Centers have been a key characteristic of North American health systems, this concept has only recently been adopted internationally. The Academic Health Center in an organized structure that began to appear in the US by the end of 1940. When the size and complexity of an AHC begins to increase, universities reorganize the health sciences in a specific campus, creating dedicated governance and administrative oversight as well as specialized clinical services 1. However, there is not a specific definition of an AHC. From a general point of view, an AHC could be public or private; it must have a medical school, an affiliated hospital and – as of recently – it should be incorporated into the same campus as other schools or courses linked to health. A generic attribute of these complex and highly ranked organizations is engagement in high-quality clinical services, deep involvement with science as well as clinical and basic research, and a permanent commitment to the formal education of health professionals at a high standard 3.

Academic Health Centers have become strategic partners for the governments of Western developed countries. Most AHCs are totally or partially funded by governments, encouraging the translation of innovation and research into products that benefit society. In the USA, academic health systems are mostly financed by private insurance, as revenues from faculty practice represent an important part of the total budget of the organizations. To complement this revenue, academic health systems usually receive public funding for research. However, as Thomas D. Giles wrote in an editorial 3, Academic Health Centers have suffered a transformation in the fundamental pillars of academic medicine, scholarly research, and teaching, which nurtured the “golden age of medicine” into what is now the “Business Age”. According to this editorial, medical students, fellows, and house officers are now oriented to the business model rather than being mostly educated in the traditional scientific model; consequently, it is challenging to recruit young physicians and researchers into traditional academic careers. The general tendency is to prefer private medicine 3.

In England, which is characterized by universal coverage of public health assistance, innovations are appearing in the traditional system of clinical practice. In the last decade, the government has launched a new enterprise in an effort to implement medical innovation and a high standard of patient care in the national system of public health coverage. The commitment from health authorities has mobilized academic and health care leaders to establish new organizational models to foster the best health care for society, integrating biomedical research with health care facilities. To pursue this innovative model, the Department of Health proposed a new national research strategy for 2006 to 2010, called “Best Research for Best Health”. The centerpiece of this proposition was to integrate the funds from NHS and R&D (National Health System and Research and Development) under the governance of the recently funded National Institute for Health Research (MNIHR) 4. England's emerging organizational models, designed as Academic Health Science Centers (AHSCs), are described as having four components: a clinical enterprise organization, academic-clinical enterprise integration, an academic enterprise organization and governance by an academic professor. In March 2009, the government officially announced the first five English AHSCs 4. Professor A. Darzi from Imperial College stated in a 2010 paper that the common term of the “university” or “teaching” hospital does not fully capture the concept of AHSC, which should only be applied to Institutions with six major characteristics:

1. Integrated governance, academic and clinical services
2. International and recognized excellence in research and clinical quality standards
3. Specific funding for research and teaching
4. Integrated leadership and career tracks in an academic environment
5. Integrated programs combining research and clinical assistance
6. A mission of innovation and commercial expertise that benefits the country's economy

However, since the Flexnerian reform more than one century ago and the creation of modern AHCs, many countries and communities are still facing unparalleled challenges in the quality of health care and clinical services. Even in developed countries, disparities in access to the system, the unaffordably high costs of health care and wide disparities in clinical outcomes are common 6.

Although many developed countries funded AHSCs in the hope that their research and innovation would increase national economic growth and improve the quality of health in local communities, some leaders are proposing new avenues for AHSCs 6. Prof. Dzau 6 stated that globally, these organizations might lead to disruptive medicine by inserting a new and transformative vision in medical education and creating a network to disseminate a culture of knowledge and innovation. Aside from creating novel drugs, devices, and technologies, AHSCs should propose effective and cheaper assistance to poor communities, particularly by providing effective preventive medicine to socially compromised population 6.

Finally, the current AAHC (Association for Academic Health Centers) was established in 1969, with an office located in Washington, DC. It represents 120 high-ranking American Academic Health Centers. In 2008, the international branch (AAHCI) was created, and in 2014, international offices were opened; international membership has increased to include 140 members from 26 countries.

Since 2010, FMUSP has been a member of the American Association of Academic Health Centers (AAHC). During the period 2014 to 2018, the Dean's Office launched a project to internationalize the Medical School, and one goal of this project was to strengthen the relationship with AAHC. In July 2017, the steering committee of the international branch of this association (AAHCI), together with AAHC governance, decided to open the first regional office in Latin America and the Caribbean. FMUSP was selected to host the office for two years, and in 2019, the commitment was renewed for two more years. In 2017, the former CEO/President of AAHC, Dr Steven A. Wartman wrote the following statement published in their press release 7: “We're pleased and honored to have a growing global presence and the privilege of supporting increasing numbers of academic health centers worldwide, AAHCI Regional Offices bring together academic health centers to promote regional activities, as well as programs and interests that are particularly germane to the area. The offices also serve to provide valuable insight for AAHC and AAHCI on key issues and challenges faced in each region”. With the launch of these new offices, AAHCI regional offices promote targeted activities and programs by

- Determining the specific interests and needs of AAHCI members located in their region
- Facilitating regional networking opportunities, educational programs, exchange of best practices, data collection and analysis, and community/regional relationships.
- Promoting the academic health center concept to governments, industry, and the public.

Our Academic Health Center is composed of a Medical School, the University Hospital (Hospital das Clínicas), residency and PhD programs. We have more than 200 research groups, courses for specialization in different areas of health, a school for technical education in health and a close collaboration with the Schools of Nursing and Public Health that are located in the same area. We believe that our academic health center is a good model for other institutions in Latin America and the Caribbean, showing that it is possible to develop a high-quality institution dedicated to heath in developing countries. Moreover, the Regional Office that we will be overseeing until 2022 will work intensively to increase the number of members affiliated, especially by offering them the opportunity to participate in regional networks to promote more effective south-to-south cooperation (a network for PhD programs has been initiated). We will also facilitate the interchange of experience with Academic Health Centers of other regions, including those from the Northern Hemisphere.
